# In Situ Metal Sulfide-Modified N/S-Doped Carbon for High-Performance Oxygen Reduction

**DOI:** 10.3390/ijms27010434

**Published:** 2025-12-31

**Authors:** Mingyuan Zhang, Jinru Wang, Caihan Zhu, Yuning Zhang, Dewei Li, Shuozhen Hu

**Affiliations:** 1 Materials Tech Laboratory for Hydrogen & Energy Storage, Ningbo Institute of Materials Technology and Engineering, Chinese Academy of Sciences, Ningbo 315201, China; zhangmingyuan@nimte.ac.cn (M.Z.);; 2 State Key Laboratory of Advanced Marine Materials, Ningbo Institute of Materials Technology and Engineering, Chinese Academy of Sciences, Ningbo 315201, China; 3 Nano Science and technology Institute, University of Science and Technology of China, Suzhou 215123, China; 4 Shanghai Institute of Optics and Fine Mechanics, Chinese Academy of Sciences, Shanghai 201800, China; ynzhang@siom.ac.cn; 5 State Key Laboratory of Chemical Engineering, East China University of Science and Technology, Shanghai 200237, China

**Keywords:** fuel cell, oxygen reduction reaction, non-noble metal catalysts, N/S co-doped, transition metal sulfides

## Abstract

Developing efficient and durable oxygen reduction reaction (ORR) catalysts is crucial for advancing fuel cell technology and sustainable energy conversion. In this study, a scalable strategy was employed to synthesize ZIF-derived nitrogen-sulfur co-doped carbon nanosheets embedded with in situ generated ZnS and Co_9_S_8_ nanoparticles. The synergistic effect of heteroatom doping and metal sulfide modification effectively modulated the electronic structure, optimized charge transfer pathways, and enhanced structural stability. The optimized catalyst exhibited a half-wave potential of 0.83 V vs. RHE, close to that of commercial 20 wt% Pt/C (0.85 V), excellent 4e^−^ ORR selectivity, and exceptional stability, with only a ~15 mV degradation after 10,000 cycles. These results demonstrate that the combination of nitrogen sulfur co-doping and in situ metal sulfide addition pro-vides an effective approach for designing highly active and durable non-precious metal catalysts for the ORR. This synthetic concept provides practical guidance for the scalable preparation of multifunctional nanomaterial-based catalysts for electrochemical energy applications.

## 1. Introduction

The continuous growth of global energy consumption is still primarily supported by fossil fuels, which, while providing reliable energy, also contribute significantly to environmental pollution and greenhouse gas emissions [1,2,3]. To achieve sustainable development, clean and renewable energy technologies have attracted increasing attention [4,5]. Among them, fuel cells stand out for their high efficiency and zero-emission operation [6]. However, the sluggish kinetics of the oxygen reduction reaction (ORR) at the cathode limit overall fuel cell performance, necessitating platinum-based catalysts to enhance reaction rates [7]. Platinum-based catalysts account for 38% to 60% of the total cost of a fuel cell stack [8,9,10,11]. The high cost and limited availability of Pt—responsible for a substantial fraction of stack cost—remain major barriers, while many non-precious metal catalysts (NPMCs) suffer from insufficient mass activity and limited durability for practical use [12,13,14,15].

Zeolitic imidazolate frameworks (ZIF-8, ZIF-67) have attracted significant interest as precursors for M–N–C catalysts due to their high nitrogen content, controllable metal centers, and porous carbon skeletons [16,17]. These ZIF-derived structures enable uniform dispersion of active sites and optimized electronic environments, making them attractive platforms for the design of highly active ORR electrocatalysts for fuel cell cathodes [18]. Heteroatom doping, particularly with nitrogen and sulfur, has proven effective in modifying the electronic structure of carbon matrices, facilitating O_2_ adsorption and charge transfer through synergistic effects [19,20,21,22,23]. Sulfur incorporation forms C–S–C and SOx–C bonds, tuning the charge density of adjacent carbon atoms and thereby enhancing ORR kinetics. Furthermore, coupling with transition metal sulfides introduces additional active centers and can strengthen the structural robustness of the catalyst [24].

Recent studies have demonstrated the promise of such hybrid systems. Peng et al. [25] reported Co_9_S_8_ nanoparticles embedded in N/S co-doped hollow carbon nanosheets, achieving a half-wave potential (E_1_/_2_) of 0.82 V and excellent bifunctional ORR/OER performance. Li et al. [26] prepared ZnS@NC catalysts by sulfidation and carbonization of ZIF-8, achieving a high starting potential of 0.94 V and a half-wave potential of 0.84 V comparable to commercial 20% Pt/C. Huang et al. [27] designed ZnS/Co-NSCNTs through the co-sulfidation of ZIF-8 and ZIF-67, where the synergistic effect of ZnS and thiophene-S species facilitated O_2_ adsorption and improved electron mobility at Co–N_x_ sites. However, despite these advances, scalable synthesis strategies that integrate multi-metal sulfides with N/S-doped carbon nanosheets while maintaining high activity and durability remain limited.

In this study, a scalable strategy was developed to synthesize ZIF-derived N/S co-doped carbon nanosheets embedded with in situ formed ZnS and Co_9_S_8_ nanoparticles. The synergistic coupling of heteroatom doping and metal sulfide modification effectively tailored the electronic structure, optimized charge transfer, and enhanced structural stability. As a result, the Co_9_S_8_/Cu-SNC catalyst exhibited an ORR half-wave potential of 0.83 V vs. RHE, comparable to that of commercial Pt/C, and demonstrated superior durability with only about 15 mV loss after 10,000 cycles and excellent methanol tolerance in 0.1 M KOH. These findings provide a practical and cost-effective route for constructing high-performance non-precious metal catalysts for next-generation fuel cells.

## 2. Results and Discussion

**Figure 1:**
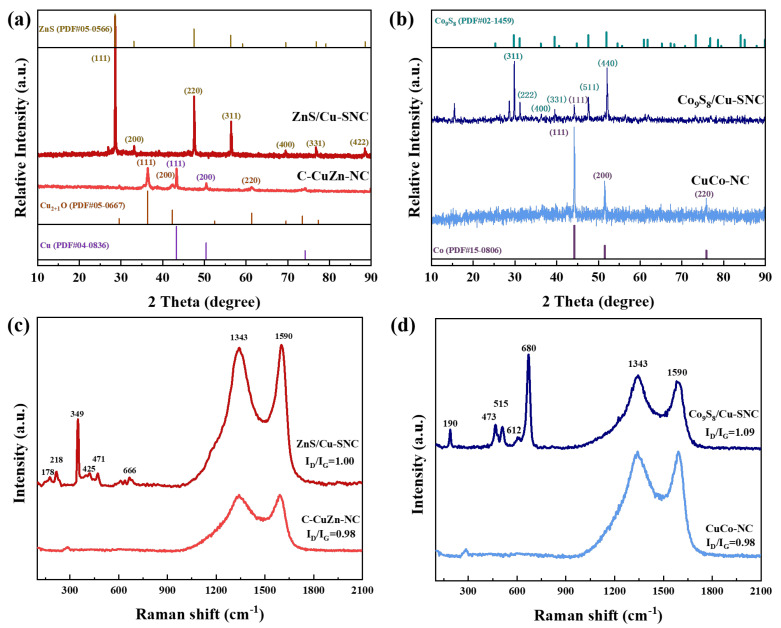
XRD patterns of (**a**) ZnS/Cu-SNC and CuZn-NC catalysts, (**b**) Co_9_S_8_/Cu-SNC and CuCo-NC catalysts; Raman spectra of (**c**) ZnS/Cu-SNC and CuZn-NC catalysts, (**d**) Co_9_S_8_/Cu-SNC and CuCo-NC catalysts.

**Figure 2:**
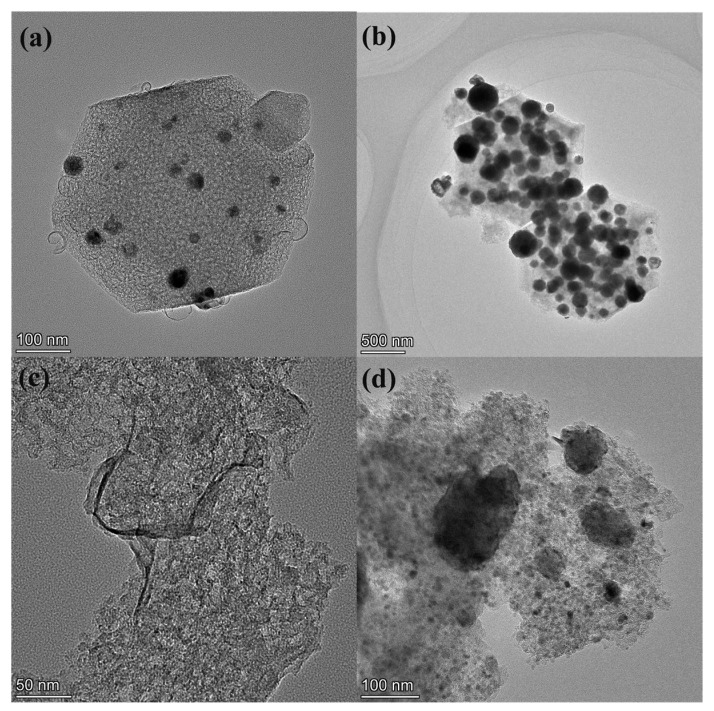
TEM spectra of (**a**) CuZn-NC, (**b**) CuCo-NC, (**c**) ZnS/Cu-SNC, and (**d**) Co_9_S_8_/Cu-SNC.

The crystal structures of CuZn-NC, ZnS/Cu-SNC, CuCo-NC, and Co_9_S_8_/Cu-SNC were tested and analyzed by XRD, and the results are shown in Figure 1a,b. It can be observed from the XRD pattern of the CuZn-NC catalyst that obvious diffraction peaks appear at 2θ = 43.3°, 50.4°, and 74.1°, which belong to the (111), (200) and (220) crystal planes of metal Cu (PDF#04-0836). In addition, characteristic peaks belonging to the (220) and (111) crystal planes of Cu_2+1_O were observed at 2θ = 61.3° and 36.4° (PDF#05-0667). The above results show that the CuZn-NC catalyst obtained by high-temperature pyrolysis of Cu@ZIF-8 contains Cu and Cu_2+1_O crystals. This is because during the high-temperature pyrolysis process, the Cu^2+^ adsorbed in the ZIF-8 framework that did not participate in the reaction can be reduced to zero-valent Cu by carbon, and part of the Cu will be oxidized by the air to form Cu oxides. The characteristic peaks observed at 2θ = 28.6°, 33.1°, 47.5°, 56.3°, 69.5°, 76.8°, and 88.5°of the ZnS/Cu-SNC catalyst are corresponding to the (111), (200), (220), (311), (400), (331), and (422) crystal planes of ZnS, respectively (PDF#05-0566). This is because during high-temperature pyrolysis, evaporated Zn reacts with S to form ZnS. When ZIF-67 was used as the precursor, the XRD spectrum of the CuCo-NC catalyst only shows characteristic peaks belonging to the metal Co (111), (200), and (220) crystal planes at 2θ = 44.2°, 51.5°, and 75.8° (PDF#15-0806). However, for the XRD spectrum of the Co_9_S_8_/Cu-SNC catalyst, it can be seen in Figure 2b that there are obvious characteristic peaks belonging to the Co_9_S_8_ (311), (222), (331), (511) and (440) crystal planes at 2θ = 29.8°, 31.2°, 39.5°, 47.6° and 52.1° (PDF#02-1459), and the sharp relative intensity of peaks indicates that the crystallization intensity is very high. During the pyrolysis of Cu@ZIF-67, the sulfurized reaction formed Co_9_S_8_ crystals, which are typical spiral crystal structures, are formed by alternating arrangement of Co and S atoms, and contain two different ring structures, Co_6_S_8_ and Co_3_S_4_. This alternating spiral structure can increase the electron transfer rate during the reaction and accelerate the reaction kinetics.

The Raman spectra of ZnS/Cu-SNC, CuZn-NC, Co_9_S_8_/Cu-SNC, and CuCo-NC, as shown in Figure 1c,d, exhibit two characteristic peaks (D and G) located at 1343 and 1590 cm^−1^, respectively. The D peak originates from structural defects and disordered edges in carbon materials, whereas the G peak arises from the in-plane vibration of sp^2^-hybridized carbon atoms, reflecting the degree of graphitization. A higher intensity ratio (I_D_/I_G_) indicates an increased defect density within the carbon skeleton. CuZn-NC and CuCo-NC, derived from the pyrolysis of ZIF-8 and ZIF-67 with nearly identical frameworks, show comparable carbon structures and defect levels. In contrast, ZnS/Cu-SNC and Co_9_S_8_/Cu-SNC exhibit slightly higher I_D_/I_G_ values after sulfur doping, suggesting that S incorporation enhances defects in carbon materials, and improves charge delocalization within the carbon lattice, consistent with Raman I_D_/I_G_ increase, which is known to facilitate ORR kinetics. Compared with CuZn-NC, ZnS/Cu-SNC displays additional Raman peaks at 178, 218, 349, 425, 471, and 666 cm^−1^. The peaks at 178, 349, and 666 cm^−1^ correspond to the transverse acoustic (TA), first-order longitudinal optical (1LO), and second-order longitudinal optical (2LO) phonon modes of ZnS, respectively. The additional features near 218, 425, and 471 cm^−1^ arise from surface vibrations of ZnS nanoparticles, attributed to symmetry breaking at the nanoscale that relaxes the q = 0 selection rule. These results confirm the formation of ZnS nanoparticles via vulcanization–sulfidation of the Cu@ZIF-8 precursor during high-temperature pyrolysis with sublimed sulfur [28]. Similarly, the Co_9_S_8_/Cu-SNC catalyst exhibits five distinct Raman peaks at 190, 473, 515, 612, and 680 cm^−1^ (as shown in Figure 2d), corresponding to the F_2_g, Eg, F_2_g, F_2_g, and A_1_g vibrational modes of Co_9_S_8_, respectively. This further verifies the formation of Co_9_S_8_ upon sulfur incorporation during pyrolysis. The consistency between Raman and XRD analyses supports that metal sulfides form concurrently with sulfur doping into the carbon matrix during high-temperature treatment.

The morphology and elemental distribution of all synthesized samples were analyzed using transmission electron microscopy (TEM), and the results are presented in Figure 2. As shown in Figure 2a,b, the CuZn-NC and CuCo-NC catalysts, derived from the pyrolysis of Cu@ZIF-8 and Cu@ZIF-67 at 900 °C, retain the rhombic dodecahedral structure of the original ZIF framework without any significant structural collapse or agglomeration. Additionally, a high density of nanoparticles is observed on the surface of the rhombic dodecahedron. Following sulfurization with sulfur powder as the sulfur source, the CuZn-NC and CuCo-NC catalysts were transformed into ZnS/Cu-SNC and Co_9_S_8_/Cu-SNC, respectively, as evidenced by their TEM images shown in Figure 2c,d. Notably, after sulfurization, the ZnS/Cu-SNC and Co_9_S_8_/Cu-SNC catalysts lose their original rhombic dodecahedral morphology and instead adopt a nanosheet-like structure, with a small quantity of nanoparticles dispersed across the surface.

**Figure 3:**
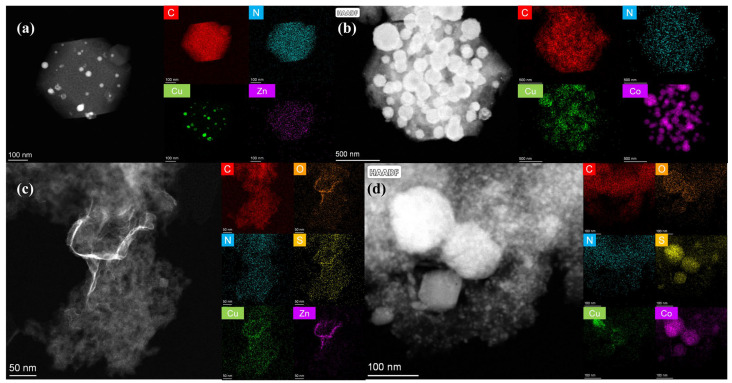
The HAADF-STEM and elemental mapping images of (**a**) CuZn-NC, (**b**) CuZn-NC, (**c**) ZnS/Cu-SNC, and (**d**) Co_9_S_8_/Cu-SNC.

Figure 3 presents the elemental mapping images corresponding to the four catalysts described above. As observed in Figure 3a, C and N elements are uniformly distributed across the rhombic dodecahedron structure, with the nanoparticles in the CuZn-NC catalyst primarily composed of metallic Cu. Additionally, the elemental distribution map reveals that the Zn content in the catalyst is minimal, which can be attributed to the volatilization of Zn during the high-temperature pyrolysis process [29]. As a result, only a small fraction of Zn is anchored by nitrogen in the organic ligand and remains within the catalyst. In Figure 3b, it is evident that the majority of nanoparticles in the CuCo-NC catalyst are composed of metallic Co. The C and N are evenly spread across the entire carbon carrier, while Cu is more uniformly distributed compared to that in the CuZn-NC catalyst. Furthermore, only distinct peaks of metallic Co are observed in the XRD spectrum of CuCo-NC, suggesting the reduction of Co^2+^ ions to metallic Co during the high-temperature calcination process, likely facilitated by carbon. In Figure 3c, the elemental distribution in the ZnS/Cu-SNC catalyst is homogeneous, with C, N, O, S, and Cu elements evenly dispersed across the nanosheet, while the Zn content is more concentrated within the folds of the nanosheet. Finally, in Figure 3d, for the Co_9_S_8_/Cu-SNC catalyst, the C, N, and O elements are uniformly distributed throughout the nanosheet structure, whereas the S and Co elements are predominantly localized in particles with diameters of approximately 100–150 nm.

**Figure 4:**
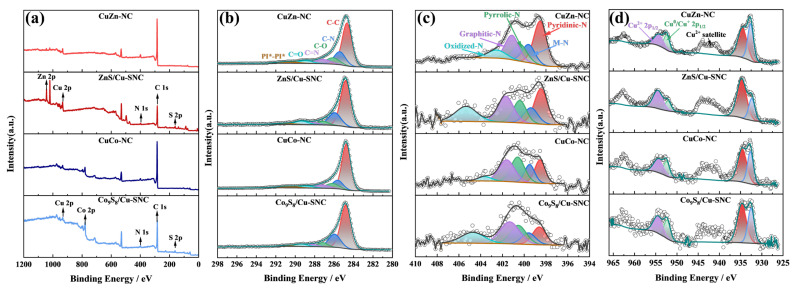
(**a**) XPS survey spectra, (**b**) high-resolution C 1s spectra, (**c**) high-resolution N 1s spectra, and (**d**) high-resolution Cu 2p spectra of CuZn-NC, CuCo-NC, ZnS/Cu-SNC and Co_9_S_8_/Cu-SNC.

The surface composition and electronic structure of the CuZn-NC, CuCo-NC, ZnS/Cu-SNC, and Co_9_S_8_/Cu-SNC catalysts were systematically investigated by X-ray photoelectron spectroscopy (XPS). As shown in Figure 4a, the characteristic signals of C, N, Cu, and Co or Zn were clearly identified in all four catalysts, confirming the presence of carbon, nitrogen, copper, and cobalt or zinc within the structures. Moreover, in the full XPS spectra of ZnS/Cu-SNC and Co_9_S_8_/Cu-SNC, an additional S 2p peak was detected, demonstrating that sulfur powder physically mixed prior to calcination was successfully incorporated into the catalysts during the high-temperature pyrolysis process. Deconvolution of the high-resolution C 1s spectrum (Figure 4b) reveals six distinct components: C–C (~284.7 eV), C–N (~285.6 eV), C–O (~286.2 eV), C=N (~287.2 eV), C=O (~288.9 eV), and π*–π* conjugated bonds (~290.8 eV). The presence of the C–N bond provides direct evidence for the successful incorporation of nitrogen atoms into the carbon framework.

The chemical states and electronic configuration of Cu species were further examined through the high-resolution Cu 2p spectra (Figure 4c). All catalysts display characteristic Cu^+^ 2p3/2 (~932.7 eV), Cu^2+^ 2p3/2 (~934.8 eV), Cu^+^ 2p1/2 (~952.4 eV), and Cu^2+^ 2p1/2 (~954.6 eV), along with satellite peaks of Cu^2+^ at ~943.0 eV and ~962.5 eV. Importantly, no discernible peak shift in Cu 2p binding energies is observed after sulfur incorporation, consistent with the XRD results. Furthermore, no crystalline phase corresponding to Cu sulfides was detected, indicating that Cu predominantly exists in the +1 and +2 oxidation states, which can be attributed to Cu oxides and Cu species coordinated with nitrogen (Cu–N structure).

The high-resolution N 1s XPS spectrum (Figure 4d) was deconvolved to yield five distinct nitrogen species: pyridinic-N (~398.5 eV), metal-N (~399.6 eV), pyrrolic-N (~400.5 eV), graphitic-N (~401.5 eV), and oxidized-N (~403.0 eV) [30]. The quantitative distribution of these N configurations is summarized in Table 1. Notably, the content of metal-N in ZnS/Cu-SNC and Co_9_S_8_/Cu-SNC catalysts decreased by approximately 3% relative to their counterparts, CuZn-NC and CuCo-NC, respectively—consistent with partial consumption of Zn and Co atoms in forming metal sulfide phases. Concurrently, sulfur incorporation significantly altered the nitrogen speciation: both sulfide catalysts exhibited a marked reduction in the relative abundance of pyridinic-N, while graphitic-N increased substantially. This suggests that sulfur doping induces electronic restructuring within the N-doped carbon matrix. Although metal-N bonds are often regarded as key active centers in M–N–C catalysts [31], the introduction of sulfur not only reorganizes the carbon scaffold but also generates metal sulfide domains, complicating activity–structure relationships. Importantly, graphitic-N has been reported to govern limiting current density and enhance electrical conductivity. In line with this, the Co_9_S_8_/Cu-SNC catalyst exhibits the highest graphitic-N content (30.91%), which correlates with an anticipated high limiting current density [32].

**Figure 5:**
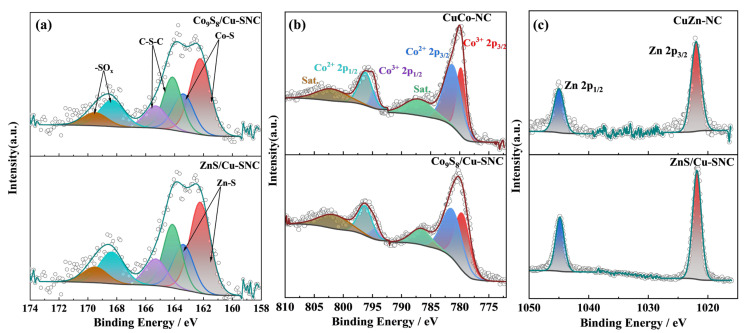
(**a**) High-resolution S 2p spectra, (**b**) High-resolution Co 2p spectra, and (**c**) High-resolution Zn 2p spectra of CuZn-NC, CuCo-NC, ZnS/Cu-SNC and Co9S8/Cu-SNC catalysts.

The high-resolution S 2p spectra were employed to elucidate the valence states of sulfur species and their interactions with both the carbon matrix and metal centres. As shown in Figure 5a, the S 2p spectrum can be deconvoluted into three major components: M–S (metal–sulfur), C–S–C (thienyl-S), and –SO_x_ species [33]. Specifically, the Co–S 2p3/2 (~161.8 eV) and 2p1/2 (~162.7 eV) peaks, together with the Zn–S 2p3/2 and 2p1/2 peaks showing negligible binding energy shifts, confirm the formation of Co sulfides in Co_9_S_8_/Cu-SNC and Zn sulfides in ZnS/Cu-SNC, respectively. Additional peaks at ~168.2 eV and ~169.9 eV are assigned to sulphate species, while those at ~163.9 eV and ~164.9 eV correspond to the 2p3/2 and 2p1/2 of thienyl-S (C–S–C). The coexistence of C–N and thienyl-S functionalities demonstrate the successful dual doping of N and S atoms into the carbon skeleton. Moreover, thienyl-S is known to modulate the charge density and spin distribution of carbon atoms, as well as interact synergistically with pyridinic-N, thereby enhancing oxygen reduction reaction (ORR) activity [34]. Quantitatively, the Co_9_S_8_/Cu-SNC catalyst exhibits a higher thienyl-S content (47.82%) compared to ZnS/Cu-SNC (41.16%), which rationalizes its superior ORR performance.

Figure 5b presents the high-resolution Co 2p spectra of CuCo-NC and Co_9_S_8_/Cu-SNC. The binding energies at ~779.6 eV (Co^3+^ 2p3/2) and ~794.5 eV (Co^3+^ 2p1/2), along with ~781.3 eV (Co^2+^ 2p3/2) and ~796.3 eV (Co^2+^ 2p1/2), indicate the coexistence of Co in both +2 and +3 oxidation states. Furthermore, the shake-up satellite features at ~786.7 eV and ~801.7 eV corroborate the presence of Co^2+^ species. Similarly, the high-resolution Zn 2p spectra of CuZn-NC and ZnS/Cu-SNC (Figure 5c) display characteristic Zn^2+^ doublet peaks at ~1021.9 eV (2p3/2) and ~1044.9 eV (2p1/2), confirming the oxidation state of Zn in the sulfide catalyst.

**Figure 6:**
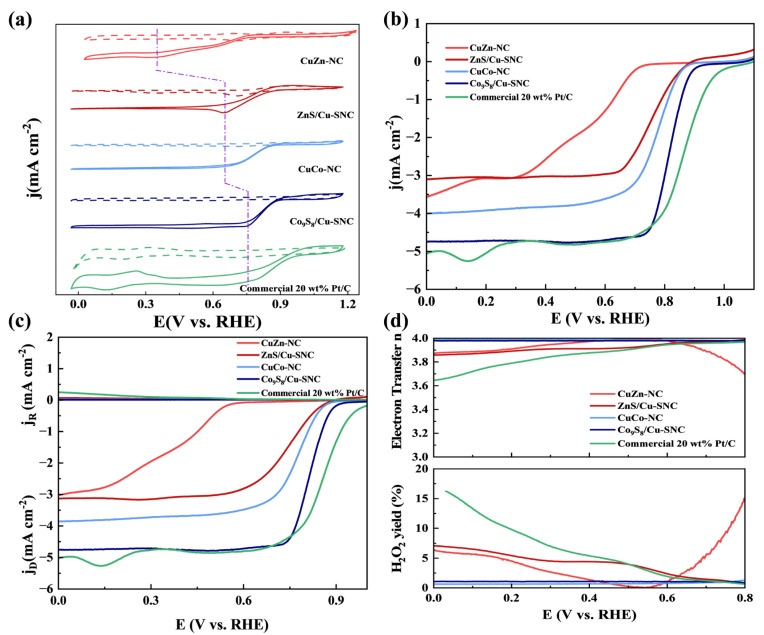
(**a**) Cyclic voltametric curves in N_2_-saturated (dashed line) and O_2_-saturated (solid line) 0.1 M KOH solutions at the scan rate of 10 mV s^−1^, (**b**) LSV curves at 1600 rpm, (**c**) The jD and jR, (**d**) H_2_O_2_% and electron transfer number calculated by RRDE curves from 0.2 V to 0.8 V of CuZn-NC, CuCo-NC, ZnS/Cu-SNC, Co_9_S_8_/Cu-SNC and commercial 20 wt.% Pt/C catalysts.

The ORR activity of CuZn-NC, CuCo-NC, ZnS/Cu-SNC, and Co9S8/Cu-SNC was evaluated using a three-electrode system with a rotating disk electrode, with a commercial 20 wt.% Pt/C as reference. As shown in Figure 6a, cyclic voltammetry (CV) curves in 0.1 M KOH saturated with N_2_ and O_2_. Compared with the test results under N_2_ atmosphere, the CV curves of the four catalysts prepared experimentally and the Pt/C catalyst in the O_2_-saturated electrolyte all revealed obvious oxygen reduction peaks, indicating that the prepared catalysts have ORR catalytic activity. Linear sweep voltammetry (LSV) was used to determine the onset potential (E_onset_) and half-wave potential (E_1/2_) of each catalyst. ZnS/Cu-SNC (E_onset_ = 0.87 V, E_1/2_ = 0.75 V) and Co_9_S_8_/Cu-SNC (E_onset_ = 0.92 V, E_1/2_ = 0.83 V) exhibit significantly higher E_onset_ and E_1/2_ than CuZn-NC (E_onset_ = 0.73 V, E_1/2_ = 0.57 V) and CuCo-NC (E_onset_ = 0.88 V, E_1/2_ = 0.77 V), approaching the performance of commercial Pt/C (E_onset_ = 1.04 V, E_1/2_ = 0.85 V). The high activity of Co_9_S_8_/Cu-SNC is the result of the highly dispersed Co-N active sites, the optimized electronic structure of S/N co-doping, the synergistic effect of Co_9_S_8_ nanoparticles, and the defect-rich carbon skeleton. These factors work together to make its half-wave potential close to that of commercial 20 wt.% Pt/C.

Rotating ring-disk electrode (RRDE) measurements assessed H_2_O_2_ selectivity and electron transfer pathway. As shown in Figure 6c, ring current densities follow the order Pt/C > CuZn-NC > CuCo-NC ≈ ZnS/Cu-SNC > Co_9_S_8_/Cu-SNC, indicating that the prepared catalysts predominantly follow a four-electron pathway. Figure 6d shows calculated H_2_O_2_ yields and average electron transfer numbers (*n*): CuZn-NC (3.7%, *n* = 3.93), CuCo-NC (3.9%, *n* = 3.92), ZnS/Cu-SNC (1.1%, *n* = 3.97), Co_9_S_8_/Cu-SNC (0.7%, *n* = 3.99), and Pt/C (5.9%, *n* = 3.87). These results demonstrate that the four experimental catalysts exhibit superior 4e^−^ selectivity compared with Pt/C, with Co_9_S_8_/Cu-SNC showing the highest ORR activity.

**Figure 7:**
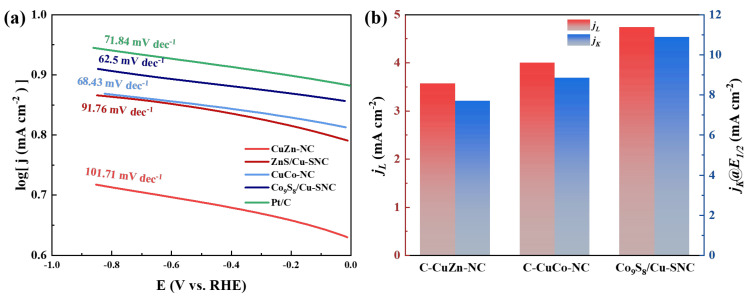
(**a**) Tafel plot comparison of the samples and (**b**) The *jL* and *jk* of CuZn-NC, CuCo-NC, ZnS/Cu-SNC, Co_9_S_8_/Cu-SNC catalysts.

The Tafel slope can be used to analyze the sensitivity of the current response to the applied potential, thereby providing information related to the rate-determining step. As shown in Figure 7a, the Tafel slopes of the four prepared catalysts and the commercial 20 wt% Pt/C catalyst are ranked from large to small as follows: CuZn-NC (101.71 mV dec^−1^) > ZnS/Cu-SNC (91.76 mV dec^−1^) > Pt/C (71.84 mV dec^−1^) > CuCo-NC (68.43 mV dec^−1^) > Co_9_S_8_/Cu-SNC (62.5 mV dec^−1^). The higher the Tafel slope value, the relatively poorer ORR kinetic reaction rate of the CuZn-NC and ZnS/Cu-SNC catalysts. However, for CuCo-NC and Co_9_S_8_/Cu-SNC, the Tafel slopes dropped significantly and were lower than those of the Pt/C catalyst. This shows that the improvement strategy for the Co_9_S_8_/Cu-SNC catalyst is successful, and some Co_9_S_8_ nanoparticles are evenly distributed on the carbon support modified by S and N. This is conducive to the adsorption and subsequent reduction of O2 molecules, and ultimately improves the ORR kinetics, resulting in a lower Tafel slope value. The larger the limiting diffusion current density, the greater the current that can pass through per unit area per unit time, and the faster the reaction kinetics. As a fuel cell cathode catalyst, it may make the fuel cell have greater power. From Figure 7b, it can be seen that the Co_9_S_8_/Cu-SNC catalyst has the largest limiting diffusion current density (4.7 mA cm^−2^), which is much higher than the other three catalysts. At the same time, it also shows the highest kinetic current density calculated at the half-wave potential. Combined with the above K-L equation and the calculation results of the Tafel slope and kinetic current density, the Co_9_S_8_/Cu-SNC catalyst exhibits better kinetic performance in the ORR process. defect-rich carbon and pyridinic/graphitic N sites increase the density and intrinsic activity of ORR active sites, which typically raises the kinetic current density (*jk*) and shifts E_1_/_2_ positively by improving O_2_ adsorption and facilitating O–O bond activation. Combining structural characterization and performance testing, defect-rich carbon and pyridinic/graphitic N sites increase the density and intrinsic activity of ORR active sites, which typically raises the kinetic current density (*jk*) and shifts E_1_/_2_ positively by improving O_2_ adsorption and facilitating O–O bond activation [35]. Sulfur doping in the carbon framework and in metal sulfides (e.g., Co_9_S_8_) modulates the local electronic structure of adjacent metal-nitrogen centers and creates new, more optimized adsorption sites for O*, OH*, and OOH*, thereby lowering the reaction barrier and resulting in a smaller Tafel slope and higher *jk* [36].

**Figure 8:**
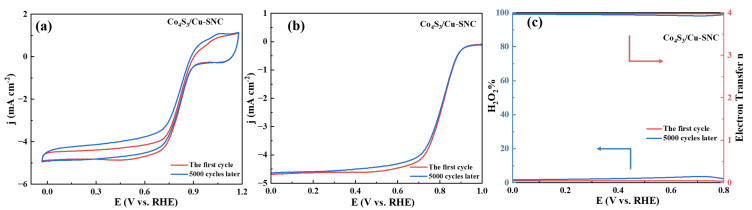
(**a**) The CV curves of Co_9_S_8_/Cu-SNC catalyst before and after10,000 cycles ADT; (**b**) LSV curves of Co_9_S_8_/Cu-SNC catalyst before and after 10,000 cycles ADT; and (**c**) H_2_O_2_% and electron transfer number of Co_9_S_8_/Cu-SNC catalyst before and after ADT, in O_2_-saturated 0.1 M KOH.

In the comprehensive evaluation of the catalyst, excellent stability is also crucial, which determines the service life of the fuel cell in commercial applications. The stability of the Co_9_S_8_/Cu-SNC catalyst was evaluated by accelerated durability testing (ADT). The ADT was performed in O_2_-saturated 0.1 M KOH by cyclic voltammetry between 0.60 and 1.00 V vs. RHE at a scan rate of 100 mV s^−1^ for 10,000 cycles, with the catalyst loading kept at ~0.4 mg cm^−2^. As can be seen from Figure 8a, the initial reduction potential of Co_9_S_8_/Cu-SNC remained basically unchanged before and after 10,000 cycles of cyclic voltammetry testing, indicating that its overpotential did not increase due to the accelerated durability test. The half-wave potential E_1/2_ decayed by only 15 mV after 10,000 cycles of cyclic voltammetry testing, which further demonstrates the excellent durability of the catalyst (Figure 8b). At the same time, we examined the catalyst 4e^−^ selectivity before and after the ADT. In the voltage window of 0−0.8 V, H_2_O_2_% only increased from 0.7% to 2.5%, while the average electron transfer number just decreased from 3.99 to 3.95. This fully confirms that after the accelerated durability test, the Co_9_S_8_/Cu-SNC catalyst still maintains very excellent 4e^−^ pathway ORR catalytic ability.

## 3. Materials and Methods

### 3.1. Materials and Chemicals

Methanol (CH_3_OH, 99%) and Zinc nitrate hexahydrate (Zn(NO_3_)_2_ • 6H_2_O, 99.99%) were purchased from Titan Chemical Co., Ltd. (Shanghai, China). Copper nitrate tri-hydrate (Cu(NO_3_)_2_ • 3H_2_O, 99.99%) and Cobalt nitrate hexahydrate (Co(NO_3_)_2_ • 6H_2_O, 99.99%) were purchased from Aladdin Biochemical Technology Co., Ltd. (Shanghai, China). Di-methylimidazole (C_4_H_6_N_2_, 99%) was purchased from Meryer Chemical Technology Co., Ltd. (Shanghai, China). Sublimed sulfur (S, 99%) was purchased from Sinopharm Chemical Reagent Co., Ltd. (Shanghai, China). Potassium hydroxide (KOH, 98%) was purchased from Macklin Biochemical Technology Co., Ltd. (Shanghai, China). Nafion^®^ solution (5 wt%) and the commercial 20 wt% Pt were purchased from Sigma-Aldrich (Shanghai, China). All the chemicals were analytical reagent grade and used without further purification.

### 3.2. Synthesis of CuZn-NC and CuCo-NC Catalyst

**Figure 9:**
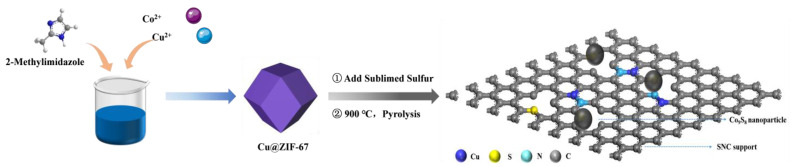
Schematic illustration of the preparation of the Co_9_S_8_/Cu-SNC catalyst.

Firstly, the preparation method similar to the traditional ZIF-8 was used. As shown in Figure 9, solution A was prepared by dissolving 3.941 g of 2-methylimidazole in 160 mL of methanol solution, and continuously and vigorously stirring until it was completely dissolved. Then, solution B was prepared by mixing and dissolving 0.873 g of zinc nitrate hexahydrate (Zn(NO_3_)_2_·6H_2_O) or 0.827 g of cobalt nitrate hexahydrate (Co(NO_3_)_2_·6H_2_O) and 0.725 g of copper nitrate trihydrate (Cu(NO_3_)_2_·3H_2_O) in 160 mL of methanol solution with vigorous stirring. In a constant temperature of 30 °C, solution B was quickly poured into solution A while stirring continuously. The mixed continued to be vigorously stirred for 8 h and allowed to stand for two hours. After the reaction, the blue or purple precipitate was separated by centrifugation and methanol washing, and then dried in vacuum at 60 °C for 12 h to obtain a white (Cu@ZIF-8) or purple solid (Cu@ZIF-67).CuZn-NC and CuCo-NC electrocatalysts were obtained by calcining Cu@ZIF-8 and Cu@ZIF-67 precursors. The specific steps were as follows: the Cu@ZIF-8 or Cu@ZIF-67 powder was fully ground and placed in a tube furnace. The temperature was raised to 900 °C at a rate of 5 °C min^−1^ under argon atmosphere, and the precursor was pyrolyzed at 900 °C for 2 h. After the sample was cooled naturally to room temperature, the black powder was collected. The final products are represented as CuZn-NC and CuCo-NC.

### 3.3. Synthesis of ZnS/Cu-SNC and Co_9_S_8_/Cu-SNC

During the calcination of Cu@ZIF-8 and Cu@ZIF-67 precursors, the S element was introduced by secondary doping. At first, sulfur powder was added and fully mixed with the ZIF materials during the grinding of Cu@ZIF-8 or Cu@ZIF-67. Subsequently, the precursor with sulfur powder was pyrolyzed at 900 °C for 2 h under an argon atmosphere, utilizing the same steps as the pyrolysis method for obtaining CuZn/NC and CuCo-NC, and the obtained black powders were named ZnS/Cu-SNC and Co_9_S_8_/Cu-SNC, respectively.

### 3.4. Structural Characterization

The morphology and microstructural features of samples were examined by transmission electron microscopy (TEM, JEM-2100, JEOL, Tokyo, Japan) High-angle annular dark-field scanning transmission electron microscopy (HAADF-STEM) coupled with energy-dispersive X-ray spectroscopy (EDS) mapping was performed on a Talos F200X microscope (Thermo Fisher Scientific, Waltham, MA, USA) to analyze the elemental distribution. The crystalline structure of the catalysts was determined by X-ray diffraction (XRD, D8 Advance, Bruker, Karlsruhe, Germany) using Cu Kα radiation (λ = 1.5406 Å, 40 kV, 15 mA). Raman spectra were recorded on a LabRAM HR confocal Raman spectrometer (Horiba J.Y., Paris, France) with a 514.5 nm excitation laser. The surface chemical states were investigated by X-ray photoelectron spectroscopy (XPS, Thermo Scientific K-Alpha^+^, Waltham, MA, USA), and all binding energies were calibrated against the C 1s peak at 284.8 eV.

### 3.5. Electrochemical Tests

All electrochemical measurements were conducted on the Biologic (SP-50e) workstation (Seyssinet-Pariset, France) by a three-electrode system at room temperature in 0.1 M KOH. The RDE or RRDE coated by catalysts served as the working electrode, an Ag/AgCl (saturated KCl) electrode as the reference electrode, and graphite as the counter electrode. All potentials shown in this study were converted to reversible hydrogen electrode (RHE) potentials according to the following formula: E (vs. RHE) = E (vs. Ag/AgCl) + 0.0592 pH + 0.2046.

The RRDE tests were performed at a ring electrode potential of 1.274 V (vs. RHE), with a scan rate of 5 mV s^−1^ and an electrode rotation rate of 1600 rpm. The number of electron transfers (*n*) and H_2_O_2_ yield (H_2_O_2_%) were calculated according to the following formulas:

(1) HO2−%=200×iRNiD+iRN

(2) n=4×iRNiD+iRN

where, iR represents the ring current collected at the Pt ring, iD represents the disk current, *n* is the number of electron transfers, and N is the current collection efficiency of the RRDE, typically taken as 0.37.

The catalytic kinetic current density (*jk*) can be calculated using the Koutecky-Levich (K-L) equation:

(3) $$\frac{1}{j}=\frac{1}{jk}+\frac{1}{jL}$$

in which, *j* is the current density, and *jk* and *jL* are the kinetic current density and limiting diffusion current density, respectively.

## 4. Conclusions

In this study, N, S co-doped carbon nanosheets loaded with transition metal sulfides have been successfully synthesized using ZIF-8 and ZIF-67 precursors with in situ Cu incorporation and secondary sulfurization. The resulting ZnS/Cu-SNC and Co_9_S_8_/Cu-SNC catalysts were characterized by TEM, XRD, Raman, and XPS, confirming the formation of uniformly distributed metal sulfide nanoparticles and effective dual N/S doping within the carbon matrix. The structural modifications induced by sulfur incorporation enhanced defects and graphitic-N content, which contributed to improved electronic conductivity and exposure of active sites. Electrochemical evaluation demonstrated that Co_9_S_8_/Cu-SNC exhibited a half-wave potential of 0.83 V vs. RHE, high limiting diffusion current density (4.7 mA cm^−2^), and near-ideal 4e^−^ selectivity with minimal H_2_O_2_ generation, approaching the performance of commercial 20 wt.% Pt/C. Furthermore, the catalyst maintained excellent durability after 10,000 ADT cycles, with only slight decreases in half-wave potential and electron transfer number. These results indicate that the synergistic combination of transition metal sulfides and N, S co-doped carbon effectively promotes oxygen adsorption, accelerates ORR kinetics, and stabilizes active sites, providing a promising design strategy for high-performance and durable non-precious metal ORR catalysts for fuel cell cathodes and related electrochemical energy conversion systems.

## Figures and Tables

**Table 1:** The content of different N species in N 1s high-resolution spectra of CuZn-NC, CuCo-NC, ZnS/Cu-SNC and Co_9_S_8_/Cu-SNC catalysts.

Sample	Pyridinic-N	M−N	Pyrrolic-N	Graphitic-N	Oxidized-N
CuZn-NC	37.08%	15.18%	15.96%	21.93%	9.84%
ZnS/Cu-SNC	27.25%	12.74%	17.02%	24.09%	18.9%
CuCo-NC	21.07%	15.74%	28.90%	29.22%	5.34%
Co_9_S_8_/Cu-SNC	17.39%	12.34%	21.73%	30.91%	17.63%

## Data Availability

The raw data supporting the conclusions of this article will be made available by the authors upon request.

## Abbreviations

The following abbreviations are used in this manuscript:

PEMFC	Proton Exchange Membrane Fuel Cells
jR	The current density of the ring electrode in the Rotating Ring-Disk Electrode
jD	The current density of the disk electrode in the Rotating Ring-Disk Electrode
*jk*	Dynamic current density
*jL*	Limit diffusion current density
ORR	Oxygen reduction reaction
RHE	Reversible Hydrogen Electrode
I_D_/I_G_	D peak and G peak intensity ratio
TA	Transverse acoustic
1LO	First-order longitudinal optical
2LO	Second-order longitudinal optical
